# The Practice, Outcome and Complications of Tracheostomy in Traumatic Brain Injury Patients in a Neurosurgical Intensive Care Unit: Surgical versus Percutaneous Tracheostomy and Early versus Late Tracheostomy

**DOI:** 10.21315/mjms2022.29.3.7

**Published:** 2022-06-28

**Authors:** Yusrina Zahari, Wan Mohd Nazaruddin Wan Hassan, Mohd Hasyizan Hassan, Rhendra Hardy Mohamad Zaini, Baharuddin Abdullah

**Affiliations:** 1 Department of Anaesthesiology and Intensive Care, School of Medical Sciences, Universiti Sains Malaysia, Kelantan, Malaysia; 2 Hospital Universiti Sains Malaysia, Universiti Sains Malaysia, Kelantan, Malaysia; 3 Department of Otorhinolaryngology, School of Medical Sciences, Universiti Sains Malaysia, Kelantan, Malaysia

**Keywords:** traumatic brain injury, tracheostomy, intensive care unit, outcome, mechanical ventilation

## Abstract

**Background:**

The tracheostomy procedure is commonly required to wean patients off the severe traumatic brain injury (TBI). This study aimed to determine the practice, outcome and complications of two techniques: i) surgical tracheostomy (ST) versus percutaneous tracheostomy (PT) and ii) two different times of procedure: early tracheostomy (ET) versus late tracheostomy (LT).

**Methods:**

This was a retrospective, cross-sectional study conducted from 1 January 2013 until 31 December 2017, involving 268 severe TBI patients who required tracheostomy during neurosurgical intensive care unit (Neuro-ICU) management. The data were obtained from their medical records.

**Results:**

When based on techniques, PT displayed a significantly shorter day of tracheostomy plan (7.0 [2.5] versus 8.3 [2.6] days; *P* < 0.001); day of execution (7.2 [2.6] versus 8.6 [2.9] days; *P* < 0.001); duration of mechanical ventilation (9.8 [3.4] versus 11.3 [3.1] days; *P* < 0.001) and duration of ICU stay (12.3 [3.7] versus 13.8 [3.5] days; *P* < 0.003) than ST. If based on timing, ET showed a significantly shorter duration of mechanical ventilation (8.8 [2.1] versus 12.9 [2.9] days; *P* < 0.001), length of ICU stay (11.4 [2.4] versus 15.2 [3.5] days; *P* < 0.001) and length of hospital stay (17.1 [3.2] versus 20.0 [4.0] days; *P* < 0.001) than LT.

**Conclusion:**

PT showed a shorter mechanical ventilation and ICU stay duration than ST. In comparison, ET showed shorter mechanical ventilation, ICU stay and hospital stay duration than LT.

## Introduction

Traumatic brain injury (TBI) is one of the top three reasons for intensive care unit (ICU) admission in Malaysia in the last decade (1). Based on data from the National Trauma Database (NTrD) 2009, 63.6% of major trauma is TBI (2). The leading causes of traumatic injuries include motor vehicle accidents (74.4%), falls from heights (12.2%) and assault (4.8%) (3). The mortality rate of TBI worldwide ranges from 5.2 to 80.73 per 100,000 populations per year (4). TBI can be classified based on Glasgow Coma Scale (GCS) into mild (GCS 13–15), moderate (GCS 9–12) and severe (GCS 8 or less) (5). Severe TBI commonly requires intubation and prolonged ventilation in the ICU, and if the GCS recovery is poor, patients commonly require tracheostomy procedure for faster weaning from mechanical ventilation and reducing the risk of ventilator-associated pneumonia (VAP) (6–7).

Tracheostomy is a procedure that creates an airway opening through the trachea at a level about two to three fingerbreadths from the sternal notch (8). The technique can be divided into surgical technique (ST) or percutaneous technique (PT). PT is commonly performed in five different techniques: i) balloon dilation; ii) guide wire dilating forceps; iii) multiple dilator; iv) single step dilation and v) translaryngeal tracheostomy (9). In our centre, we use single step dilation technique using Tracoe® tracheostomy set. The ST is usually performed by an otorhinolaryngology (ORL) surgical team. Alternatively, PT is performed by an anaesthesiologist or intensivist (8). Despite the evolution of percutaneous dilatational techniques, ST remains the gold standard for patients with airway emergencies and abnormal anatomy (10). The PT accesses the trachea with a needle using the Seldinger technique, followed by insertion of a guidewire then tracheostomy tube over the guidewire after dilatation (11). PT generally requires less time to perform, is more cost-effective, is typically performed sooner and reduces the risk of stoma infection (12–13). The indications for tracheostomy after TBI include an inability to wean from invasive mechanical ventilation, lack of protective airway reflexes, diminished respiratory drive and difficulty managing secretions (7, 14). Previous studies have discussed the benefits of tracheostomy in the weaning of TBI patients, which include shorter mechanical ventilation and, thus, ICU stay duration (7, 15–16). Another study also revealed that early tracheostomy (ET) was associated with a reduction in long-term mortality (17).

Our institution is the main tertiary centre for neurosurgery in the east coast of Malaysia, which receives approximately 400–600 cases per year, whereby about 35% are related to TBI. There is a dedicated neurosurgical intensive care unit (Neuro-ICU) that can simultaneously hold 12 ventilated patients. Our centre practices both tracheostomy techniques for indicated neurosurgical patients (particularly TBI patients) and the PT set used at our centre was the Tracoe® PT set. However, no studies have been conducted on local data reviewing our tracheostomy practices that can help improve our clinical practices. Our study aimed to determine the practice, outcomes and complications of two techniques, ST versus PT; and two different time of procedure, ET versus late tracheostomy (LT) for TBI patients in our Neuro-ICU. We hypothesised that PT might show earlier time of tracheostomy procedure, less complications and better in outcome than ST. On the other hand, the timing of ET might also show lower complications and better in outcome than LT.

## Methods

Patients at the age of 18 years old–65 years old who were admitted to the Neuro-ICU with severe TBI and required tracheostomy during the study period were screened; those who met the criteria without cervical spine injuries, other vital organs injuries and previous history of tracheostomy, were enrolled in the study. A total of 268 patients were selected for the study between 1 January 2013 and 31 December 2017.

Their medical records were obtained from the medical records unit and were subsequently reviewed in terms of demographic data, injury details, surgical procedure history, tracheotomy process, tracheostomy techniques, tracheostomy complications and clinical outcomes based on the duration of mechanical ventilation, length of ICU stay, length of hospital stay, GCS score on discharge, Glasgow Outcome Scale (GOS) at 6 months, GOS at 1 year, the incidence of VAP and hospital mortality.

Sample size calculation was done using G*Power 3.1 software based on multiple variables found in previous studies (14, 18–21). The significance level (α) was 0.05, power was 0.8, with an allocation ratio of 1:3 (PT: open tracheostomy). This ratio was decided based on the pilot study. If based on the mean mechanical ventilation duration with an effect size of 0.4, the total sample size was 254. Calculating the mean length of ICU stay with an effect size of 0.44, the total sample size was 220; for the length of hospital stay with the effect size of 0.5, the total sample size was 172. After incorporating the 5% withdrawal rate, the final sample size was 268 with a ratio of 1:3 (PT:ST).

Data were analysed using SPSS software version 26.0 (IBM SPSS Inc., NY, USA). Descriptive statistics were used to describe general demographic data and the practice of tracheostomy. Independent *t*-test was used to analyse the numerical data, and the Pearson’s chi-squared test was used to compare categorical data between the two different groups based on the timing and different techniques. *P* < 0.05 was considered statistically significant.

## Results

**Figure 1:**
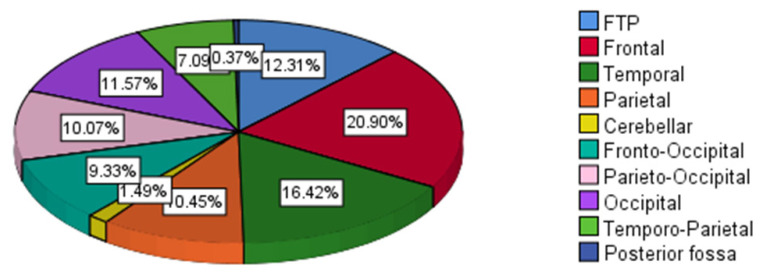
Distribution of the sites of injury

**Figure 2:**
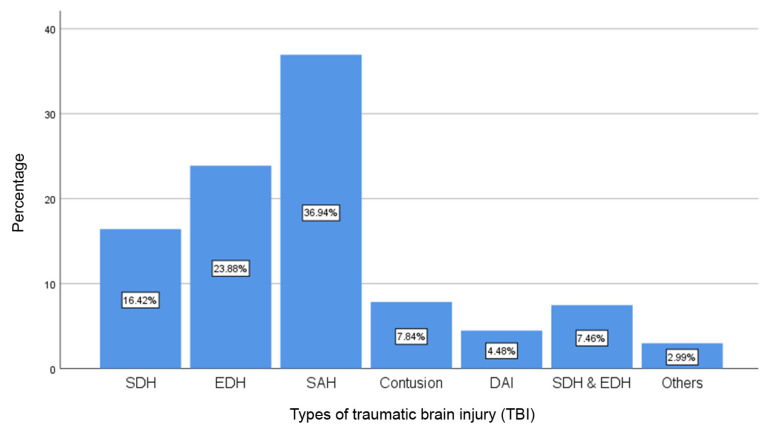
Distribution of the types of TBI Notes: SDH = subdural haematoma; EDH = epidural haematoma; SAH = subarachnoid haemorrhage; DAI = diffuse axonal injury

**Figure 3:**
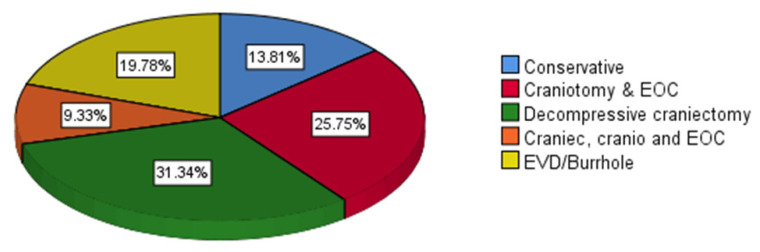
Distribution of the types of neurosurgery

The mean age was 34.5 (14.4) years old with predominantly male patients (220 [82.1%]) (Table 1). The mean of age was not significant between ST and PT groups (34.2 [14.0] versus 35.3 [15.4]; *P* = 0.585) and was also not significant between ET and LT (34.3 [13.6] versus 34.6 [15.1]; *P* = 0.846) (Tables 2 and 5). The site of most injury was the frontal lobe (56 [20.9%]) (Figure 1) and the most common type of injury was a subarachnoid haemorrhage (SAH) (99 [36.9%]) (Figure 2). The mean (SD) of GCS score upon admission was 5.7 (1.7) and the most common type of surgical procedure was decompressive craniectomy (84 [31.3%]) (Figure 3). In terms of techniques, 201 patients (75.0%) underwent ST and 67.0 (25.0%) underwent PT. With regards to the timing of tracheostomy, 126.0 (47.0%) underwent ET and 142.0 (53.0 %) underwent LT (Table 1).

When comparing the two tracheostomy techniques, PT showed a significantly earlier day of tracheostomy plan (7.0 [2.5] versus 8.3 [2.6] days; *P* < 0.001) and day of tracheostomy execution (7.2 [2.6] versus 8.6 [2.9] days; *P* < 0.001) than ST. PT also showed a significantly shorter duration of mechanical ventilation (9.8 [3.4] versus 11.3 [3.1] days; *P* < 0.001) and duration of ICU stay (12.3 [3.7] versus 13.8 [3.5] days; *P* < 0.003) than ST (Tables 2 and 3). There were no differences in term of demographic, other outcome parameters and complications (Table 4).

If based on timing of tracheostomy, ET showed significantly shorter day of decision (4.9 [1.0] versus 8.7 [2.4] days; *P* < 0.001), day of referral to ORL surgical team (5.0 [0.9] versus 8.8 [2.3] days; *P* < 0.001), day of tracheostomy plan (5.9 [1.0] versus 9.9 [2.2] days; *P* < 0.001), day of execution (6.0 [0.9] versus 10.3 [2.6] days; *P* < 0.001), duration of mechanical ventilation (8.8 [2.1] versus 12.9 [2.9] days; *P* < 0.001), duration of ICU stay (11.4 [2.4] versus 15.2 [3.5] days; *P* < 0.001) and duration of hospital stay (17.1 [3.2] versus 20.0 [4.0] days; *P* < 0.001) than LT (Table 5 and 6). However, LT showed significantly better GCS score at discharge (9.8 [2.6] versus 8.3 [2.7]; *P* < 0.001) and GOS at 6 months (3.7 [0.8] versus 3.3 [0.8]; *P* < 0.001) (Table 6). There were no differences in term of demographic, other outcome parameters and complications (Table 7).

In terms of tracheostomy complications, there were no significant differences between PT and ST (Table 4) as well as ET and LT (Table 7). There were also no significant differences in specific complications between the groups (Tables 4 and 7).

## Discussion

Our study was planned to review the tracheostomy practice demographics in TBI patients and compare the two different tracheostomy techniques and tracheostomy timing. Our study revealed that PT had a significantly earlier day of tracheostomy plan and day of tracheostomy execution than ST. The outcome of PT was also better in terms of mechanical ventilation and ICU stay duration. All PTs were carried out bedside by the managing anaesthesiology team. This could be the contributing factor for the faster execution due to the lack of necessity to arrange an operation theatre and waiting time. Additionally, this was due to the lack of waiting time for the ORL surgical team to review the PT group. In terms of tracheostomy timing, ET showed a significantly shorter day of decision, day of referral to the ORL surgical team, day of tracheostomy plan, day of tracheostomy execution, duration of mechanical ventilation, duration of ICU stay and duration of hospital stay than LT. In terms of patient outcomes, ET also showed a significantly shorter duration of mechanical ventilation, length of ICU stay and length of hospital stay than LT. However, the LT group had a significantly better GCS score at discharge and a GOS score at 6 months.

Many previous studies have been conducted on the roles of tracheostomy in TBI. The TracMan study concluded that ET did not improve the mortality rate in TBI patients, which is similar to our study (21). Other studies concluded that ET reduced the duration of mechanical ventilation, ICU stay and hospital stay (6, 22). Some other studies suggested that it also reduced the risk of VAP (16). Apart from that, patients on tracheostomy had lower sedation requirements and better patient comfort (21). Most studies define ET less than 5, 6, 8, or 10 days after admission to the definitive care centres (18, 23–24). Our centre does not have a specific guideline regarding the indications for tracheostomy in TBI. Hence, making it difficult to determine the right timing for tracheostomy in our TBI patients. Our study chose day 7 as the cut-off point, taking into consideration that patients have completed the resuscitation phase and all necessary surgical interventions. This duration also provides ample time for our neurosurgeons to prognosticate the patient and counsels next of kin for consent. Further prospective, randomised controlled trials are needed to accurately assess the cut-off point. The proportion of patients in our study who had ET or LT is comparable. Our study significantly supported ET, as the outcomes of our patients in terms of duration of mechanical ventilation, length of ICU stay and length of hospital stay were shorter. If the outcomes were based on GCS at discharge and GOS at 6-month, LT was better. One of the reasons might be due to better in baseline GCS of LT than ET at admission (6.4 [1.4] versus 4.6 [1.4]; *P* < 0.001). However, the longer term GOS at 1-year was not significant.

Several clinical studies have compared PT and ST, suggesting comparability between both techniques and even the potential superiority of PT over ST (13, 25–26). Our study showed limited complications associated with both techniques. However, each arm of the technique in our study was not equally balanced, with the ratio of 1:3 (ST:PT). This ratio was due to the higher percentage of ST, as this study was a retrospective study from the year 2013 until 2017 when PT was introduced and the availability of PT sets was based on the hospital budget because the cost of PT set is higher than the ST set. The anaesthesiologists and postgraduate trainees familiar with the PT technique were most likely few during that period. We found no significant association of complication between these two techniques. Other resources suggested that PT was associated with reduced stomatitis, scarring, obstruction, accidental decannulation, difficulty changing tubes and major bleeding (27). There are also statistically significant differences with respect to postoperative bleeding rates, surgical bleeding, and mortality based on the systematic review (28). The longitudinal follow-up suggested that the delayed complications, which included clinically significant tracheal stenosis, were similar in both (29). However, our study did not identify the long-term complications of tracheostomy, such as tracheal stenosis, tracheomalacia, trachea-oesophageal fistula, dislodged tracheostomy and obstructed tracheostomy due to incomplete documentation on tracheostomy care. PT has gained widespread acceptance due to its great ease of execution (29). Both tracheostomy techniques should be safe with proper and adequate training. Some biases confound the limited, mainly retrospective, available data on this issue. Our results suggest that the current, local medical practices influence the decision to perform a tracheostomy, along with the ethical and legal implications context, clinical expertise, and costs relating to the procedure and equipment, replicating past findings in the general ICU population. The optimal indications for tracheostomy remain uncertain as policies and clinical practice vary among different centres (30). Hence, a randomised controlled trial (RCT) is very much needed on this topic.

## Limitations

As a retrospective study, we could not avoid problems regarding missing, conflicting and illegible data. The observational nature of the study only allows to report associations and cannot test the causal relationships between factors and tracheostomy practice (7).

## Conclusion

PT showed better outcomes in TBI patients, with a shorter duration of mechanical ventilation and ICU stay than ST. In terms of timing of tracheostomy, ET showed better outcomes than LT with a shorter duration of mechanical ventilation, ICU stay and hospital stay. However, LT showed better GCS scores at discharge and GOS scores at 6 months. Our study showed the importance of early timing of tracheostomy with percutaneous technique for TBI patient in order to get better outcome parameters. We hope a future prospective RCT study on the complications and outcomes of the early timing of tracheostomy between PT and ST.

## Figures and Tables

**Table 1:** General demographic data

Parameters	Descriptive statistic (*n* = 268)
Age (years old)	34.5 (14.4)
Gender	
Male	220.0 (82.1%)
Female	48.0 (17.9%)
GCS on admission	5.7 (1.7)
Time related to surgery	
Time interval of surgery from the onset of injury (hours)	8.2 (4.2)
Time interval of surgery from Emergency Department admission (hours)	1.7 (2.2)
Duration of surgery (hours)	6.2 (4.6)
Timing of tracheostomy	
Early (≤ 7 days)	126.0 (47.0%)
Late (> 7 days)	142.0 (53.0 %)
Techniques of tracheostomy	
Open/surgical technique	201.0 (75.0%)
Percutaneous technique	67.0 (25.0%)
Process of tracheostomy	
Day of tracheostomy decision (days)	6.9 (2.6)
Day of review by ORL surgical team (days)	7.0 (2.6)
Time interval of referral to review by ORL surgical team (hours)	2.1 (3.3)
Day of plan (days)	8 (2.6)
Day of execution (days)	8.3 (2.9)

Notes: All numerical data are expressed in mean (SD) and all categorical data in *n* (%)

**Table 2:** Demographic data based on techniques of tracheostomy

Parameters	Surgical tracheostomy (*n* = 201)	Percutaneous tracheostomy (*n* = 67)	*P*-value
Age (years old)	34.2 (14.0)	35.3 (15.4)	0.585^a^
Gender			
Male	160 (79.6%)	60 (89.6%)	> 0.950^b^
Female	41 (20.4%)	7 (10.4%)	
GCS on admission	5.8 (1.7)	5.4 (1.7)	0.148^a^
Site of brain injury			0.903^c^
Fronto-temporo-parietal	24 (11.9%)	9 (13.4%)	
Fronto-occipital	18 (9.0%)	7 (10.4%)	
Parieto-occipital	19 (9.5%)	8 (11.9%)	
Temporo-parietal	14 (7.0%)	5 (7.5%)	
Frontal	43 (21.4%)	13 (19.4%)	
Temporal	30 (14.9%)	14 (20.9%)	
Parietal	23 (11.4%)	5 (7.5%)	
Occipital	26 (12.9%)	5 (7.5%)	
Cerebellar	3 (1.5%)	1 (1.5%)	
Posterior fossa	1 (0.5%)	0 (0.0%)	
Types of injury			0.038^c^
SDH	34 (16.9%)	10 (14.9%)	
EDH	53(26.4%)	11 (16.4%)	
SAH	74 (36.8%)	25 (37.3%)	
SDH and EDH	14 (7.0%)	6 (9.0%)	
Contusion	10 (5.0%)	11 (16.4%)	
DAI	11 (5.5%)	1 (1.5%)	
Others	5 (2.4%)	3 (4.5%)	
Type of surgery			0.418^c^
Decompressive craniectomy	58 (28.9%)	26 (38.8%)	
Craniotomy and evacuation of clots	56 (27.9%)	13 (19.4%)	
Craniectomy, craniotomy and evacuation of clots	20 (10%)	5 (7.5%)	
Burr hole and external ventricular drainage	41 (20.4%)	12 (17.9%)	
No surgery-Conservative management	26 (12.8%)	11 (16.4%)	
Time related to surgery			
Time interval of surgery from the onset of injury (hours)	8.3 (4.4)	7.9 (3.5)	0.553a
Time interval of surgery from the Emergency Department admission (hours)	3.7 (2.1)	4.0 (2.6)	0.305^a^
Duration of surgery (hours)	6.1 (4.6)	6.4 (4.6)	0.681^a^
Process of tracheostomy			
Day of decision (days)	7.0 **(**2.6)	6.5 (2.6)	0.157 a
Day of referral to ORL surgical team (days)	7.1(2.6)	6.7 (2.4)	0.300 a
Time taken by ORL surgical team to review (hours)	2.1 (3.3)	1.7 (1.0)	0.758^a^
Day of plan (days)	8.3 (2.6)	7.0 (2.5)	*< 0.001^a^
Day of execution (days)	8.6 (2.9)	7.2 (2.6)	*< 0.001^a^

Notes: All numerical data are expressed in mean (SD) and all categorical data in *n* (%);

a Independent *t*-test;

b Pearson’s chi-squared test;

c Fisher exact test;

SDH = subdural haematoma; EDH = epidural haematoma; SAH = subarachnoid haemorrhage; DAI = diffuse axonal injury

**Table 3:** Outcomes based on tracheostomy techniques

Parameters	Surgical tracheostomy (*n* = 201)	Percutaneous tracheostomy (*n* = 67)	*P-*value
Duration of mechanical ventilation (days)	11.3 (3.1)	9.8 (3.4)	*0.001^a^
Duration of ICU stay (days)	13.8 (3.5)	12.3 (3.7)	*0.003^a^
Duration of hospital stay (days)	18.9 (3.7)	17.9 (4.3)	0.080^a^
GCS at discharge	9.3 (2.7)	8.7 (2.9)	0.100^a^
GOS at 6 months	3.6 (0.8)	3.3 (0.8)	0.082^a^
GOS at 1 year	3.5 (1.3)	3.2 (1.4)	0.170^a^
Incidence of VAP			
Yes	25.0 (12.4%)	12.0 (17.9%)	0.861^b^
No	176.0 (87.6%)	55.0 (82.1%)	
Hospital mortality			
Yes	36.0 (17.9%)	11.0 (16.4%)	0.421^b^
No	165.0 (82.1%)	56.0 (83.6%)	

Notes: All numerical data are expressed in mean (SD) and all categorical data in *n* (%);

a Independent *t*-test;

b Pearson’s chisquared test

**Table 4:** Complications of tracheostomy between two techniques

	Surgical tracheostomy (*n* = 201)	Percutaneous tracheostomy (*n* = 67)	*P*-value
Presence of complications			
Yes	32 (15.9%)	13 (19.4%)	0.509^b^
No	169 (84.1%)	54 (80.6%)	
Specific complications			
Bleeding	5 (2.5%)	2 (3.0%)	0.788^c^
False track	1 (0.5%)	1 (1.5%)	
Hypoxaemia	4 (2.0%)	1 (1.5%)	
Difficult procedure	13 (6.5%)	5 (7.5%)	
Infection	3 (1.5%)	1 (1.5%)	
Pneumothorax	5 (2.5%)	1 (1.5%)	
Others	1 (0.5%)	2 (3.0%)	

Notes: All categorical data are expressed in *n* (%);

b Pearson’s chi-squared test;

c Fisher exact test

**Table 5:** Demographic data based on timing of tracheostomy

Parameters	Early tracheostomy (*n* = 126)	Late tracheostomy (*n* = 142)	*P*-value
Age (years old)	34.3 (13.6)	34.6 (15.1)	0.846^a^
Gender			
Male	103 (81.7%)	117 (82.4%)	0.508^b^
Female	23 (18.3%)	25 (17.6%)	
GCS on admission	4.6 (1.4)	6.6 (1.4)	*< 0.001^a^
Site of brain injury			0.823^c^
Fronto-temporo-parietal	16 (12.7%)	17 (11.9%)	
Fronto-occipital	13 (10.3%)	12 (8.5%)	
Parieto-occipital	15 (11.9%)	12 (8.5%)	
Temporo-parietal	9 (7.1%)	10 (7.0%)	
Frontal	20 (15.9%)	36 (25.3%)	
Temporal	22 (17.5%)	22 (15.5%)	
Parietal	14 (11.1%)	14 (9.9%)	
Occipital	15 (11.9%)	16 (11.3%)	
Cerebellar	2 (1.6%)	2 (1.4%)	
Posterior fossa	0 (0%)	1 (0.7%)	
Types of injury			0.313^c^
SDH	19 (15.1%)	25 (17.6%)	
EDH	33 (26.2%)	31 (21.8%)	
SAH	50 (39.6%)	49 (34.6%)	
SDH and EDH	6 (4.8%)	14 (9.9%)	
Contusion	12 (9.5%)	9 (6.3%)	
DAI	3 (2.4%)	9 (6.3%)	
Others	3 (2.4%)	5 (3.5%)	
Types of surgery			0.511^c^
Decompressive craniectomy	38 (30.2%)	46 (32.4%)	
Craniotomy and evacuation of clots	29 (23.0%)	40 (28.2%)	
Craniectomy, craniotomy and evacuation of clots	15 (11.9%)	10 (7.0%)	
Burr hole and external ventricular drainage	24 (19.0%)	29 (20.4%)	
No surgery-Conservative management	20 (15.9%)	17 (12.0%)	
Time related to surgery			
Time interval of surgery from the onset of injury (hours)	8.1 (3.7)	8.3 (4.5)	0.617^a^
Time interval of surgery from the Emergency Department admission (hours)	3.9 (2.4)	3.7 (2.1)	0.442^a^
Duration of surgery (hours)	6.6 (5.0)	5.8 (4.3)	0.229^a^
Process of tracheostomy			
Day of decision (days)	4.9 (1.0)	8.7 (2.4)	*< 0.001^a^
Day of referral to ORL surgical team (days)	5.0 (0.9)	8.8 **(**2.3)	*< 0.001^a^
Time taken by ORL surgical team to review (hours)	1.8 (1.4)	2.3 (4.2)	0.250^a^
Day of plan (days)	5.9 (1.0)	9.9 (2.2)	*< 0.001^a^

Notes: All numerical data are expressed in mean (SD) and all categorical data in *n* (%). Statistical analysis:

a Independent *t*-test;

b Pearson’s chi-squared test;

c Fisher exact test;

SDH = subdural haematoma; EDH = epidural haematoma; SAH = subarachnoid haemorrhage; DAI = diffuse axonal injury

**Table 6:** Outcomes based on timing of tracheostomy

Parameters	Early tracheostomy (*n* = 126)	Late tracheostomy (*n* = 142)	*P*-value
Duration of mechanical ventilation (days)	8.8 (2.1)	12.9 (2.9)	*< 0.001^a^
Duration of ICU stay (days)	11.4 (2.4)	15.2 (3.5)	*< 0.001^a^
Duration of hospital stay (days)	17.1 (3.2)	20.0 (4.0)	*< 0.001^a^
GCS at discharge	8.3 (2.7)	9.8 (2.6)	*< 0.001^a^
GOS at 6 months	3.3 (0.8)	3.7 (0.8)	*< 0.001^a^
GOS at 1 year	3.1 (1.4)	3.6 (1.3)	0.156^a^
Incidence of VAP			
Yes	18 (14.3%)	19 (13.4%)	0.268^b^
No	108 (85.7%)	123 (86.6%)	
Hospital mortality			
Yes	25 (19.8%)	22 (15.5%)	0.063^b^
No	101 (80.2%)	120 (84.5%)	

Notes: All numerical data were expressed in mean (SD) and all categorical data in *n* (%);

a Independent *t*-test;

b Pearson’s chi-squared test

**Table 7:** Complications of tracheostomy between two techniques

Parameters	Early tracheostomy (*n* = 126)	Late tracheostomy (*n* = 142)	*P*-value
Presence of complications			
Yes	21 (16.7%)	24 (16.9%)	0.959^b^
No	105 (83.3%)	108 (83.1%)	
Specific complications			
Bleeding	1 (0.8%)	6 (4.2%)	0.548^c^
False track	2 (1.6%)	0 (0.0%)	
Hypoxaemia	3 (2.4%)	2 (1.4%)	
Difficult procedure	9 (7.1%)	9 (6.3%)	
Infection	2 (1.6%)	2 (1.4%)	
Pneumothorax	3 (2.4%)	3 (2.1%)	
Others	1 (0.8%)	2 (1.4%)	

Notes: All categorical data are expressed in *n* (%);

b Pearson’s chi-squared test;

c Fisher exact test
